# Epidemic infectious gastrointestinal illness aboard U.S. Navy ships deployed to the Middle East during peacetime operations – 2000–2001

**DOI:** 10.1186/1471-230X-6-9

**Published:** 2006-02-25

**Authors:** Mark S Riddle, Bonnie L Smoak, Scott A Thornton, Joseph S Bresee, Dennis J Faix, Shannon D Putnam

**Affiliations:** 1 Department of Preventive Medicine and Biometrics, Uniformed Services University of the Health Sciences, Bethesda, MD, USA; 2 Division of Preventive Medicine, Walter Reed Army Institute of Research, Silver Spring, MD, USA; 3 Navy Environmental and Preventive Medicine Unit #6, Pearl Harbor, HI, USA; 4 National Center for Infectious Disease, Centers for Disease Control and Prevention, Atlanta, GA, USA; 5 Navy Environmental and Preventive Medicine Unit #5, San Diego, CA, USA; 6 U.S. Naval Medical Research Unit #2, Jakarta, Indonesia

## Abstract

**Background:**

Infectious gastrointestinal illness (IGI) outbreaks have been reported in U.S. Navy ships and could potentially have an adverse mission impact. Studies to date have been anecdotal.

**Methods:**

We conducted a retrospective analysis of weekly reported disease and non-battle injury health data collected in 2000 – 2001 from 44 U.S. Navy ships while sailing in the 5^th ^Fleet (Persian Gulf and nearby seas).

**Results:**

During this period, 11 possible IGI outbreaks were identified. Overall, we found 3.3 outbreaks per 100 ship-weeks, a mean outbreak duration of 4.4 weeks, and a mean cumulative ship population attack rate of 3.6%. Morbidity, represented by days lost due to personnel being placed on sick-in-quarters status, was higher during outbreak weeks compared to non-outbreak weeks (p = 0.002). No clear seasonal distribution was identified.

**Conclusion:**

Explosive outbreaks due to viruses and bacteria with the potential of incapacitating large proportions of the crew raise serious concerns of mission impact and military readiness.

## Background

Persons living or working in closed settings, such as nursing homes, prisons, hospitals, or daycare centers, are at elevated risk of infectious gastrointestinal illness (IGI) [1,2]. In addition, IGI outbreaks have been found to occur on both civilian cruise ships and military vessels [3,4], with *Norovirus *being the most common pathogen identified [4]. Outbreaks in closed settings like these are often difficult to prevent and control because the agents may have multiple modes of transmission, low infectious doses, and a large reservoir of susceptible persons (due to the short-lived immunity and multiple strain types) [5]. For instance, *Norovirus *outbreaks may involve transmission by consumption of contaminated food or water, direct person-to-person, airborne droplets of vomitus and contaminated environmental surfaces [2,6].

The disease burden of IGI is large. For instance, in the United States, *Norovirus *alone estimated to account for 23 million cases of acute gastroenteritis each year, and two-thirds of all foodborne gastroenteritis cases [7]. Furthermore, the economic burden of these illnesses has been particularly significant in the cruise ship industry, where intensive time and resources have been spent to prevent, identify and mitigate these outbreaks [8-10]. Whereas the economic burden on the cruise ship industry may be considerable, the importance of IGI in military settings affects mission impact and readiness. A recent study describing illness among U.S. Marine Corps personnel during the early phase of combat in Iraq found that IGI were the leading cause of clinical visits, and nearly one-fourth of these could be attributed to *Norovirus *[11]. Descriptions of IGI outbreaks on single U.S. Navy vessels have been published [12-15] including anecdotal reports of mission impact, but descriptive epidemiology is lacking. Using a retrospective study design analyzing weekly reporting data that are routinely collected for disease and non-battle injury (DNBI) surveillance, we describe the epidemiology of possible IGI outbreaks on ships deployed to the Persian Gulf region during a one-year period. These results are compared to findings reported among similar surveys of the cruise ship industry.

## Methods

We performed an analysis on shipboard reports of disease and non-battle injury (DNBI) to identify possible outbreaks of infectious gastroenteritis on ships deployed to the U.S. Navy 5^th ^Fleet from October 2000 through September 2001. Beginning in February 2000, the U.S. Naval Forces Central Command (COMUSNAVCENT) instituted a weekly DNBI incident morbidity surveillance system for all ships within its 'Area of Responsibility' (AOR) which constitutes waters and littoral areas of the Arabian Gulf, Gulf of Aden, Gulf of Oman, Red Sea, Arabian Sea and northwest Indian Ocean. This required each ship to submit a weekly aggregate DNBI report (standardized spreadsheet format) to COMUSNAVCENT Fleet Surgeon's office. The Fleet Surgeon's office collated all reports and forwarded them to the Navy Environmental and Preventive Medicine Unit #7 (NEPMU-7) located in Sicily, Italy. Each week, NEPMU-7 personnel conducted trend analysis, compiled a summary and reported back to the Fleet Surgeon's office any significant changes in DBNI rates. Integrity checks were conducted on all data to ensure validity that included logic checks of rates to measure consistency with previously reported denominators and with numerators for preceding weeks. Due to ship deployment dynamics, vessels often entered and exited the Persian Gulf region during the study period. While each ship keeps track of its own trends in DNBI, the particular command to where it reports its weekly summary depends on what AOR to which it is currently assigned. Therefore, complete reporting for all weeks that a ship was deployed was not available.

DNBI reporting standards define an IGI clinic visit as the initial visit of all diagnoses consistent with infection of the intestinal tract, including any type of diarrhea, gastroenteritis, "stomach flu", nausea/vomiting, or similar syndrome based on the physician's diagnosis. This category does not include non-infectious intestinal diagnoses, such as hemorrhoids, ulcers, or similar syndromes. Subsequent visits for the same illness are not recorded in subsequent DNBI reports; however, future clinic visits associated with new episodes would be recorded for any given individual. Distinct episodes were defined according the judgment of the clinical provider.

The primary outcome, mean weekly initial visit rate per 1000 sailors for the IGI category, was estimated for all ships during the 52-week period using Poisson regression modeling DNBI initial visit counts with person-weeks as exposure (weekly total force of the reporting unit) and estimating exact (Clopper-Pearson) 95% confidence intervals. In addition, because an individual ship could provide more than one weekly report during the surveillance period the assumption of independence among observations could be violated and was addressed by adding a correction for repeated measures by specifying the individual ship unit as a cluster in the model [16]. Possible IGI outbreaks were defined as episodes for which an individual ship reported rates that exceeded the upper limit of the 95% confidence interval for all ship-weeks of observation combined during two or more consecutive weeks. Consecutive outbreaks were discriminated if IGI rates fell below the 95% confidence level for two or more consecutive weeks between subsequent outbreaks.

No standard methodology exists to define what constitutes an outbreak in this population and setting. Based on infectious disease epidemiology, particularly in the area of diarrhea and vomiting illness of military importance, we chose an outbreak definition that would be specific to viral gastroenteridities by agents such as norovirus (high risk of person-to-person transmission and incapacitating illness). Outbreaks attributed to these viruses have been described to persist beyond one week among shipboard settings [3,12,13,17-19]. Possible outbreaks that met this definition were further evaluated for every week of observation available, and the duration of the outbreak was determined to include any visits the week prior to and the week after the period when rates exceeded the upper limit threshold. The inclusion of antecedent and subsequent cases in the cumulative case total and outbreak duration was used to account for the lead-in and tail periods which occur in these types of person-to-person outbreaks. Cumulative attack rates (AR) were calculated based on summing all IGI visits over the average reported shipboard population during the possible outbreak period. These possible outbreaks were further described in relation to duration of outbreak, ship type, and season. Though all ships are unique in design and function, for the purpose of this study, in addition to describing the particular class of ship (e.g. Aircraft Carrier, Amphibious Assault, Combat Support, Frigate, Destroyer, Cruiser), ship type was collapsed based on size defined as large (on average ≥ 1000 person) and small (<1000). This roughly breaks down into carriers and amphibious assault ships (large ships) versus others (small ships). Based on known climatologic features of the area of operation (Persian Gulf), calendar year was dichotomized into two seasons: summer (extremely hot), lasting April to October and winter (relatively mild), lasting from November to March.

In addition, we evaluated differences in morbidity by summing aggregative rates of sick-in-quarters (SIQ) (a medical disposition given to those who are not allowed to return to work for up to 72 hours), hospital admissions and lost work-days restricting analysis to only the large ships during periods of possible outbreaks compared to periods when no outbreak was occurring. Data on morbidity measurements including days SIQ and hospital admissions were compiled weekly at the aggregate level for all ships reporting. Therefore, comparisons of morbidity at the individual ship level could not be assessed. The restriction of this analysis to large ships was due to the aggregate nature of the data as the small number of visits associated with outbreaks on small ships would not be able to be distinguished from relatively high background visit rates of larger ships. Therefore, to assess whether there was differential morbidity during possible outbreak weeks compared to non-outbreak weeks, we included only possible outbreak periods and non-outbreak periods during which outbreaks occurred on large ships.

Statistical testing of differences in rates between different ship sizes was conducted using incidence-rate ratio estimation with exact confidence interval estimation in Stata Version 8 (College Station, TX). Statistical significance was set at p < 0.05.

## Results

**Figure 1:**
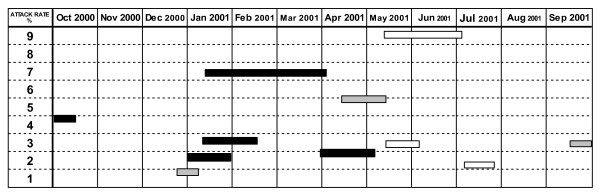
Possible infectious GI outbreaks among ships deployed to the Persian Gulf AOR during October 2000 – September 2001.  Destroyer  Aircraft Carrier  Amphibious Assault/Combat Support

Weekly DNBI reports from a total of 44 ships, accounting for 331 ship-weeks (mean 7.5 weeks/ship), were received during the deployment period. This represented approximately 29% of a ships' total 6-month deployment time. The remaining time of ships' deployments were accumulated during transit in and out of the CENTCOM AOR and were not available for review. During the study period, 1,351 visits for IGI were reported during 339,153 person-weeks of observation resulting in an IGI clinic visits summary rate for all ships of 4.0 per 1000 person-weeks (Poisson Exact 95% C.I.: 3.8, 4.3). Using the upper 95% C.I. as an upper control limit, we identified 11 possible outbreaks among 10 ships (Figure 1). Overall, mean outbreak duration was 4.4 weeks (Poisson Exact 95% C.I.: 2.6, 6.2) and the mean cumulative ship population AR was 3.6% (Poisson Exact 95% C.I.: 3.3, 3.8).

There was differential distribution of possible outbreaks on different types of ships (Table 1); outbreaks occurred on four of the six ship classes (frigates and cruisers did not have any episodes meeting the outbreak definition). Outbreak rates were higher for the larger ships with 6.6 outbreaks per 100 ship-weeks, compared to the smaller ships with 2.6 outbreaks per 100 ship-weeks, but this difference was not statistically significant (Incidence-rate ratio 2.5, 95% exact CI 0.5–9.9). In large ships (three carriers and one amphibious assault ship), the cumulative AR was 3.7% with a mean duration of 3.8 weeks compared to the smaller ships with cumulative AR of 3.6% and a mean duration of five weeks (data not shown). The months of January and May were found to have the most number of outbreaks (figure 1).

There were 611 IGI visits during non-outbreak weeks (n = 36 weeks) and 777 visits during outbreak weeks (n = 15 weeks). Based on analysis of aggregated data, there were a total of eight hospital admissions due to IGI during the study period, two of which occurred during a week when there was a possible outbreak on one of the ships and six admissions during a week when there was no outbreak activity recorded. This equates to approximately 9.8 hospital admissions per 1000 IGI visits during non-outbreak weeks compared to 2.6 hospital admissions per 1000 IGI visits during outbreak weeks (Poisson, p = 0.047). Additionally, there were 213 SIQ days given during non-outbreak weeks compared to 350 SIQ days given during outbreak weeks. This equates to 45 days of SIQ for every 100 IGI visits during an outbreak week compared to 34.9 days of SIQ for every 100 IGI visits in non-outbreak weeks (Poisson, p = 0.002).

## Discussion

Our analyses indicate that IGI outbreaks are common occurrences aboard U.S. Navy ships in this region. To the best of our knowledge, this is the first study to estimate the incidence of IGI outbreaks among deployed U.S. Navy ships. We found an overall incidence of 33.2 outbreaks per 1000 ship-weeks (Poisson 95% CI 16.6 – 59.5). A recent epidemiologic study published on IGI outbreaks among vacation cruise ships (median cruise duration of one week) found an outbreak frequency of 6.3 outbreaks per 1000 cruises (ship-weeks) during 1990 – 1995, and was reduced to 3.7 outbreaks per 1000 cruises during 1996–2000 [20]. This decrease may have been attributable to vigilance by public health and industry officials in control of person-to-person spread of illness among crew and passengers and environmental disinfection [21]. Cruise ships and Navy ships are different environments with different populations; therefore, comparisons of incidence may be misleading and should be avoided. The high rate of outbreaks among U.S. Navy ships may be explained by younger age (higher risk-taking population), higher risk ports visited by U.S. Navy ships, much longer time at sea, and/or the methods we used to define outbreaks.

The cumulative ARs in this study are similar to rates previously reported (SA Thornton, personal communication), but much lower than rates described in other IGI outbreak investigations among Navy ships [3,12,13,15]. Among 15 *Norovirus*-confirmed outbreaks, Thornton found a mean cumulative AR of 5.3%, slightly higher than what we found. This is likely due to the fact that his study used active surveillance methodology, while we relied on passive reporting. We also found cumulative attack rates that were lower than those reported among cruise ships. In a recent account of six reported outbreaks among five cruise ships during July 1 2002 to December 2002, attack rates based on sick-call visits ranged between 2.5 – 11.5% of all shipboard persons (median 6.1) [22]. A number of reasons could explain the differences in attack rates including, differences among shipboard health seeking behavior and the limitation of the surveillance window within this study possibly resulting in under-capture of all visits associated with a given outbreak. Despite finding lower attack rates, eight of eleven of the possible outbreaks we described equaled or exceeded the 3% attack rate that is used to trigger an outbreak in the cruise ship industry. As others have pointed out, the AR based on medical clinic visits likely underestimates the total number of cases associated with these outbreaks [21]. For example in one cruise ship outbreak it was found that while 8% of the passengers reported to sick call with acute gastroenteritis (AGE) symptoms, 41% met a case definition for AGE during a subsequent epidemiologic investigation (75% of passengers surveyed) [22]. In our study, we were unable to ascertain the full extent of IGI due to the passive surveillance methods used by the DNBI reporting system.

The outbreak definition we used is novel and may or may not compare equally to the Vessel Sanitation Program (VSP) classification. The VSP defines outbreaks if = 3% of the passengers and/or crewmembers develops AGE symptoms. This cutoff, developed by the CDC, was based on previous data of ship-associated outbreaks where the incidence of gastrointestinal illness was found to be = 1% of 92% of cruises and = 3% on 3% of cruises [23]. Our outbreak cut-point was based on retrospective analysis of a cohort of U.S. Navy ships deployed to the region over a one-year period. Only prospective evaluation of such a method with attempts to identify etiologic causes of these outbreaks can inform whether this method is sensitive and specific.

Outbreaks were found throughout the year, which is inconsistent with previous reports of IGI outbreaks among cruise ships which have demonstrated a winter-spring predominance and where *Norovirus *is implicated in 69% of these outbreaks [24]. It is an assumption that the outbreaks in our study are due to *Norovirus*-related disease, since a surveillance report among investigated GI outbreaks aboard large U.S. Navy ships identified *Norovirus *in 4 out of 4 which submitted stool specimens for testing *Norovirus*-confirmed outbreaks [25]. In addition, at least one of the eleven possible outbreaks identified in our study was found to be associated with *Norovirus *based on a concurrent surveillance study being conducted (SA Thornton, personal communications). The lack of a seasonality of outbreaks in our study could be explained by a number of reasons. In this equatorial region, while a season of warm and cold can be delineated, differentiation between seasons are very different than temperate climates, thus a seasonal distribution for these viruses may not be found [26-28]. Furthermore, these ships often acquire their outbreaks during visits to other equatorial parts of the world and arrive in the CENTCOM AOR with an outbreak already underway.

We also found that larger ships had more frequent outbreaks than smaller ships (6.6 per 100 ship-weeks vs. 2.6 per 100 ship-weeks, respectfully). One explanation is that larger ships often transport operational forces during deployments, with multiple embarkations and debarkations, thereby possibly introducing new enteric pathogens to shipboard personnel. It is also possible that there were differences in port visit schedules (e.g., increased number, longer duration, etc.) between the different ship classes that could account for increased risk of outbreaks. A common theme among a number of case reports has been that crowding is likely to play a role in these outbreaks [3,12,13]. One recent report published in Navy Medicine details a shipboard outbreak possibly due to viral gastroenteritis and suggests that communal toileting facilities and population density may contribute the increased risk of outbreaks [29]. Lastly, the case definition that was utilized may have resulted in fewer outbreaks associated with smaller ships, as perhaps with smaller ships outbreaks would have been recognized sooner and control measures more easily implemented. While cumulative ARs appear to be similar among the different ship classes, the duration of outbreaks appear to be longer on the smaller ships. The reason for this finding is not clear and may be due to chance or different population density and transmission dynamics among different shipboard architectures and/or populations. Differential duration of surveillance windows between large and small ships does not explain this finding either as most ships types were under observation on average 7 – 9 weeks, with only destroyers (small ship) with a shorter average surveillance window of 6.7 weeks.

While our analysis of morbidity associated with these possible outbreaks was hindered due to the aggregate nature of the data, we did find an increase in the amount of SIQ given during weeks when there were outbreaks, compared to weeks when there were no outbreaks. However, there was less hospitalization. This finding suggests that there may be a difference in severity of illnesses during outbreaks, which results in more days lost (SIQ), but requires fewer hospitalizations. The higher rate of SIQ given during outbreak weeks may also be reflective of a control strategy for IGI outbreaks, whereby ill persons are effectively removed from the workplace for 48–72 hours during the period where infectious agent shedding may be very high. Thus, the assertion of higher morbidity during outbreaks as measured by rates of SIQ may be confounded. The estimation of work days lost may be an underestimate as Whittaker *et al*. reported that during a large IGI outbreak aboard an aircraft carrier, only patients requiring treatment in the clinic were given SIQ status [14]. Thus, an undercounting of work-time lost due to illness may occur during large outbreaks. Due to the aggregate nature of this data and low numbers of hospitalizations, efforts should be made to confirm this differential morbidity before drawing any definite conclusions.

The methodology utilized in this study to define IGI outbreaks is novel and has several limitations. DNBI surveillance data collected and reported at the aggregate level is used for the purpose of tracking trends and identifying possible clusters across a broad range of clinical syndromes grouped into non-specific disease and injury categories. While this design appears useful for its intended purpose, the aggregate nature, both across individuals of each reporting ship, and across specific clinical syndromes within broad disease categories (e.g. catch-all IGI category), limits the inferences one can draw compared to more traditional individual unit of observation based epidemiological studies. Therefore, the results of this study should be put in context, and the derived estimates of particular outbreak attributes (e.g. outbreak rates, population attack rates, outbreak durations, and disease morbidity comparisons) may not be comparable to other epidemiological studies which use more traditional outbreak investigation designs. Specifically, the definition of IGI visits exceeding an upper 95% confidence (among all ships reporting) for two consecutive weeks would likely result in missing shorter outbreaks (decreased sensitivity) which are probably more commonly attributable to other common bacterial causes and a point source mode of transmission. The choice of this definition was for purposes of specificity to detect viral gastroenteritidies which can appear from a point-source introduction but tend to demonstrate a person-to-person mode of transmission with longer outbreak durations. In addition, this choice of definition may bias towards increasing the duration estimates of the described outbreaks. However, to balance this is the observation that many outbreaks were not followed to complete resolution due to the surveillance window for a particular ship ending due to its movement out of the surveillance system, thus, resulting in a possible bias towards shortening the length of a given outbreak. Furthermore, it was a limitation of our data that the incidence measurements made were only based on denominator ship-time within the CENTCOM AOR. Future studies directed at the entire deployment period should be conducted to accurate describe the true rate of these possible outbreaks.

## Conclusion

This study is a preliminary investigation to describe possible IGI outbreaks aboard U.S. Navy ships deployed to the Middle East. Despite the limitations of this methodology in defining possible outbreaks aboard U.S. Navy ships, the findings complement numerous anecdotal reports of IGI outbreaks. This is the first study that has estimated the rate of outbreaks and should serve to prompt further studies using methodology specific for the purpose of detecting, measuring and attributing cause to shipboard IGI outbreaks. We show that IGI outbreaks are common among shipboard personnel and that further studies are needed to ascertain etiology, scope, as well as contributing factors that increase outbreak risk and morbidity. In addition to sea-based studies, epidemiologic studies are also needed among land-based deployed military personnel to assess the impact of outbreak-associated IGI and to differentiate risk factors that may be specific to sea-going vessels. Whatever the cause of these possible shipboard IGI outbreaks, the epidemic potential, combined with the associated morbidity, suggests an immediate need for research and development of rapid identification assays, outbreak intervention strategies, and prophylaxis and treatment modalities where troop readiness and mission capabilities are at stake.

## Competing interests

The author(s) declare that they have no competing interests.

## Authors' contributions

MSR carried out the data collection, analysis and assisted in drafting the manuscript. BLS participated in the development of the study idea, design of the study, assisted with analysis and provided critical review. SAT assisted with data collection and helped with drafting the manusctipt. JLB assisted with manuscript development, manuscript writing, and critical review. DJF assisted with data collection, analysis and manuscript development. SDP participated in study idea, data analysis and helped to draft the manuscript. All authors read and approved the final manuscript.

## Pre-publication history

The pre-publication history for this paper can be accessed here:



## Figures and Tables

**Table 1:** Characteristics of possible outbreaks by ship class in the 5^th ^fleet during October 2000 – September 2001

**Ship Type**	**Average shipboard population**	***n *ships**	**Ship weeks**	***n *possible outbreaks**	**Mean duration (weeks)**^1^	**Cumulative attack risk (%)**	**Ship Type AR**^2 ^**(per 100 ship-weeks)**
Carrier	4951	5	45	3	2.7	3.3	6.7
Amphibious assault	669	7	56	2	5.0	7.5	3.5
Cruiser	384	6	50	0	na	na	na
Destroyer	321	18	120	5	5.4	3.8	4.2
Combat Support	551	3	22	1	(3.0)	(1.6)	4.5
Frigate	237	5	38	0	na	na	na

1. calculation based on only those ships within the class with outbreaks

2. Rate of possible outbreaks per ship-week of observation time

na = not applicable
